# Hepatocyte Growth Factor Increases Vascular Endothelial Growth Factor-A Production in Human Synovial Fibroblasts through c-Met Receptor Pathway

**DOI:** 10.1371/journal.pone.0050924

**Published:** 2012-11-28

**Authors:** Yu-Min Lin, Yuan-Li Huang, Yi-Chin Fong, Chun-Hao Tsai, Ming-Chih Chou, Chih-Hsin Tang

**Affiliations:** 1 Institute of Medicine, Chung Shan Medical University, Taichung, Taiwan; 2 Department of Orthopedic Surgery, Taichung Veterans General Hospital, Taichung, Taiwan; 3 Department of Biotechnology, College of Health Science, Asia University, Taichung, Taiwan; 4 Department of Orthopaedic Surgery, China Medical University Hospital, Taichung, Taiwan; 5 School of Chinese Medicine, China Medical University, Taichung, Taiwan; 6 Department of Medicine and Graduate Institute of Clinical Medical Science, China Medical University, Taichung, Taiwan; 7 Department of Pharmacology, School of Medicine, China Medical University, Taichung, Taiwan; 8 Graduate Institute of Basic Medical Science, China Medical University, Taichung, Taiwan; University of Nebraska Medical Center, United States of America

## Abstract

**Background:**

Angiogenesis is essential for the progression of osteoarthritis (OA). Hepatocyte growth factor (HGF) is an angiogenic mediator, and it shows elevated levels in regions of OA. However, the relationship between HGF and vascular endothelial growth factor (VEGF-A) in OA synovial fibroblasts (OASFs) is mostly unknown.

**Methodology/Principal Findings:**

Here we found that stimulation of OASFs with HGF induced concentration- and time-dependent increases in VEGF-A expression. Pretreatment with PI3K inhibitor (Ly294002), Akt inhibitor, or mTORC1 inhibitor (rapamycin) blocked the HGF-induced VEGF-A production. Treatment of cells with HGF also increased PI3K, Akt, and mTORC1 phosphorylation. Furthermore, HGF increased the stability and activity of HIF-1 protein. Moreover, the use of pharmacological inhibitors or genetic inhibition revealed that c-Met, PI3K, Akt, and mTORC1 signaling pathways were potentially required for HGF-induced HIF-1α activation.

**Conclusions/Significance:**

Taken together, our results provide evidence that HGF enhances VEGF-A expression in OASFs by an HIF-1α-dependent mechanism involving the activation of c-Met/PI3K/Akt and mTORC1 pathways.

## Introduction

Osteoarthritis (OA) is a chronic joint disorder characterized by slow progressive degeneration of articular cartilage, subchondral bone alteration, and variable secondary synovial inflammation. In response to macrophage-derived proinflammatory cytokines such as interleukin (IL)-1β and tumor necrosis factor-α (TNF-α), OA synovial fibroblasts (OASFs; the most abundant cells in OA joints) produce chemokines that promote inflammation, cartilage degradation, and neovascularization via activation of angiogenesis factors such as vascular endothelial growth factor-A (VEGF-A) [1], [2]. It has been reported that human inflammatory synovial fibroblasts including: OASF and rheumatoid arthritis (RA) SF induced angiogenesis through VEGF mediated pathway [3]. Therefore, SF mediated VEGF expression and angiogenesis play critical roles in the progression of OA and RA.

VEGF-A is a heparin binding, dimeric glycoprotein that induces the proliferation and migration of endothelial cells to form new vessels, and increases the penetration and extravagation of plasma macromolecules [4], [5]. VEGF-A has been shown to play an important role in wound healing, embryonic development, growth of certain solid tumors, and ascites formation [6]. Recently several reports demonstrated that VEGF-A was also implicated in the pathogenesis of OA [7], [8]. Treatment with a soluble form of the Flt-1 (VEGF-A receptor 1) significantly attenuated disease severity in arthritis [6], [9]. Therefore, anti-angiogenesis may be a novel therapy for OA treatment.

Hepatocyte growth factor (HGF) was identified in the early 1980s [10], [11] and was subsequently determined to be a heterodimeric molecule composed of an alpha and beta chain [12]. The importance of HGF in organ development is demonstrated by HGF null mutation mice, which exhibit embryonic lethality [13]. HGF exhibits strong angiogenic properties through its ability to induce expression of vascular endothelial growth factor, another angiogenic factor, but also has angiogenic properties of its own [14]. Recent studies have shown that the HGF plays a multifunctional role in OA cartilage and synovium [15], [16]. The complex biological action of HGF is mediated through the protooncogene c-Met, a transmembrane tyrosine kinase cell surface receptor, expressed on a multitude of cells including chondrocytes, synovial fibroblasts, and endothelial cells [17].

Hypoxia-inducible factor (HIF) is a heterodimeric transcription factor composed of the basic helix-loop-helix-Per-Arnt-Sim-domain, containing the proteins HIF-1α and arylhydrocarbon receptor nuclear translocator (HIF-1β) [18]. The availability of HIF-1 is determined primarily by HIF-1α, which is regulated at the protein level in an oxygen-sensitive manner, in contrast to HIF-1α, which is stably expressed [19], [20]. During normoxia, HIF-1α is efficiently degraded through the von Hippel-Lindau-dependent ubiquitin-proteasome pathway [20]. Under hypoxia, HIF-1α protein is markedly stabilized, translocates to the nucleus, and heterodimerizes with HIF-1β. The HIF-1α and HIF-1β complex can then bind to hypoxia response elements (HREs) located in gene promoters to regulate transcription of VEGF-A, erythropoietin, and glycolytic enzymes that enhance cellular adaptation to hypoxia [21]. Recently, the expression of VEGF-A via the activation of the phosphoinositide 3-kinase (PI3K), Akt, and mTORC1 pathway has also been shown to be mediated by HIF-1α [22], [23].

Angiogenesis is essential for the development, growth, and progression of OA [7]. VEGF-A is a potent angiogenic factor that is pivotal in the OA pathogenesis. Although a role for HGF in VEGF-A production has been implicated in some cell types, the signaling pathway for HGF in VEGF-A production in synovial fibroblasts has not been extensively studied. In this study, we explored the intracellular signaling pathway involved in HGF-induced VEGF-A production in human synovial fibroblasts. The results show that HGF and c-Met interaction activates PI3K, Akt, mTORC1, and HIF-1α pathways, leading to up-regulation of VEGF-A expression.

**Figure 1:**
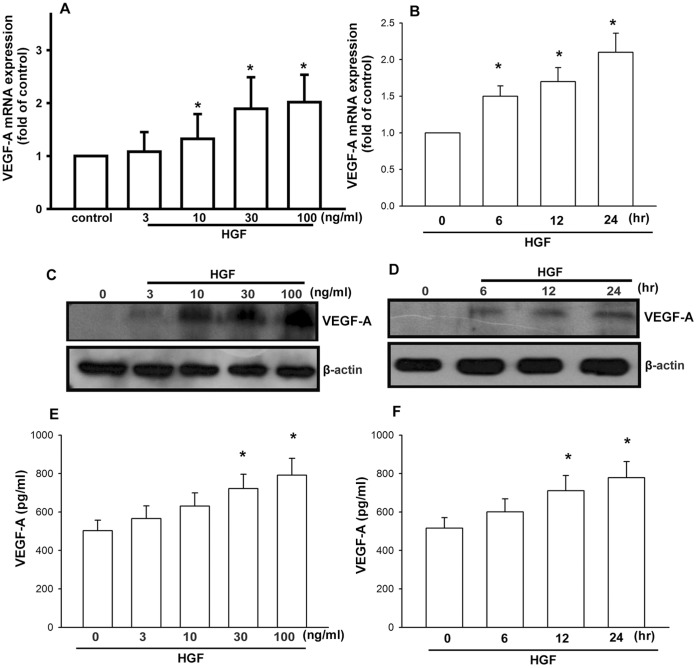
HGF stimulates concentration- and time-dependent increases in VEGF-A production. OASFs were incubated with HGF (3–100 ng/ml) for 24 h (A) or with HGF (30 ng/ml) for 6, 12, or 24 h (B), and VEGF-A mRNA was examined by qPCR. (C–F) OASFs were incubated with HGF (3–100 ng/ml) for 24 h or with HGF (30 ng/ml) for 6, 12, or 24 h, and VEGF-A protein expression was examined by Western blotting (whole cells lysate) and ELISA (medium). Results are expressed as the mean ± S.E. *, *p*<0.05 compared with control; #, *p*<0.05 compared with HGF-treated group.

**Figure 2:**
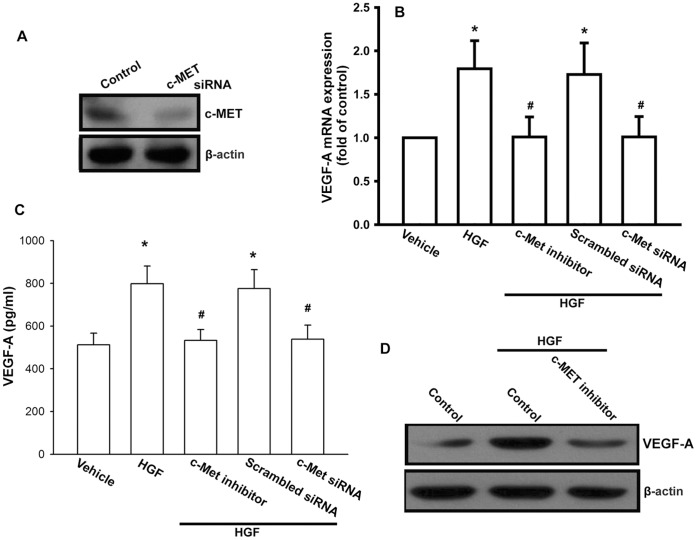
The c-Met receptor is involved in HGF-mediated VEGF-A production. (A) OASFs were transfected with c-Met siRNA for 24 h, and c-Met expression was examined by Western blotting. (B–D) OASFs were pretreated with the c-Met inhibitor (3 µM) for 30 min or transfected with c-Met siRNA for 24 h followed by treatment with HGF for 24 h, the VEGF-A expression was examined by qPCR, Western blotting, and ELISA. Results are expressed as the mean ± S.E. *, *p*<0.05 compared with control; #, *p*<0.05 compared with HGF-treated group.

## Materials and Methods

### Materials

Anti-mouse and anti-rabbit IgG-conjugated horseradish peroxidase, rabbit polyclonal antibodies specific for β-actin, PCNA, c-Met, p-p85α(Tyr467), p85α, p-Akt1(Ser473), Akt1, p-mTORC1(Ser2448), mTORC1, p-S6K(Thr389), HIF-1α, HIF-1β, and the small interfering RNAs (siRNAs) against c-Met, mTORC1, and a control for experiments using targeted siRNA transfection (each consisting of a scrambled sequence that does not lead to specific degradation of any known cellular mRNA) were purchased from Santa Cruz Biotechnology (Santa Cruz, CA). The recombinant human HGF and VEGF-A enzyme immunoassay kit were purchased from R&D Systems (Minneapolis, MN, USA). The p85α and Akt (Akt K179A) dominant negative mutant and pHRE-luciferase construct were gifts from Dr. W.M. Fu (National Taiwan University, Taipei, Taiwan). The pSV-β-galactosidase vector and luciferase assay kit were purchased from Promega (Madison, WI). All other chemicals were obtained from Sigma-Aldrich (St. Louis, MO).

### Cell Cultures

The study protocol was approved by the Institutional Review Board of China Medical University Hospital, and all subjects gave informed written consent before enrollment in this study. Human synovial fibroblasts were isolated using collagenase treatment of synovial tissues obtained from knee replacement surgeries of 33 patients with OA. Fresh synovial tissues were minced and digested in a solution of collagenase and DNase. Isolated fibroblasts were filtered through 70-µm nylon filters. The cells were grown on plastic cell culture dishes in 95% air/5% CO_2_ in RPMI 1640 (Life Technologies) that was supplemented with 20 mM of HEPES and 10% heat-inactivated FBS, 2 mM glutamine, 100 U/ml penicillin, and 100 µg/ml streptomycin (pH adjusted to 7.6). Fibroblasts from passages four to nine were used for the experiments [24], [25].

**Figure 3:**
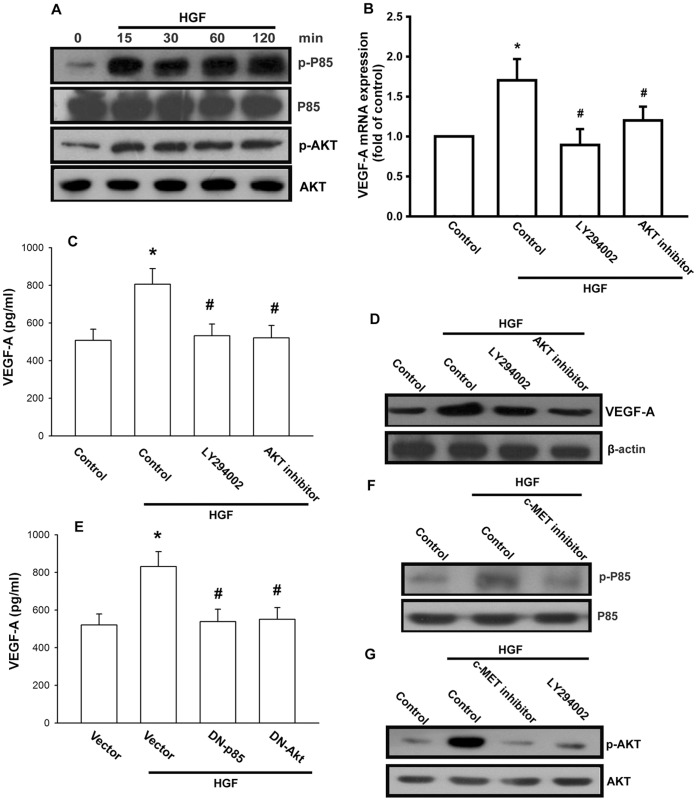
The PI3K/Akt signaling pathway is activated in response to HGF treatment of synovial fibroblasts. (A) OASFs were incubated with HGF for indicated time intervals, and p85 and Akt phosphorylation was examined by Western blotting. (B–D) OASFs were pretreated for 30 min with Ly294002 (10 µM) or Akt inhibitor (10 µM) followed by treatment with HGF for 24 h, the VEGF-A expression was examined by qPCR, Western blotting, and ELISA. (E) OASFs were transfected with p85 or Akt mutant followed by stimulation with HGF for 24 h, the VEGF-A expression was examined by ELISA. OASFs were pretreated for 30 min with c-Met inhibitor (F) or c-Met inhibitor and Ly294002 for 30 min (G) followed by stimulation with HGF for 15 min, and p85 and Akt phosphorylation was determined by Western blotting. Results are expressed as the mean ± S.E. *, *p*<0.05 compared with control; #, *p*<0.05 compared with HGF-treated group.

### Measurement of VEGF-A Production

Human synovial fibroblasts were cultured in 24-well culture plates. After reaching confluency, cells were treated with HGF (30 ng/ml) and then incubated in a humidified incubator at 37°C for 24 h. To examine the downstream signaling pathways involved in HGF treatment, cells were pretreated with various inhibitors for 30 min before addition of HGF (30 ng/ml). After incubation, the medium was removed and stored at −80°C until the assay was performed. VEGF-A in the medium was assayed using VEGF-A enzyme immunoassay kits, according to the procedure described by the manufacturer.

**Figure 4:**
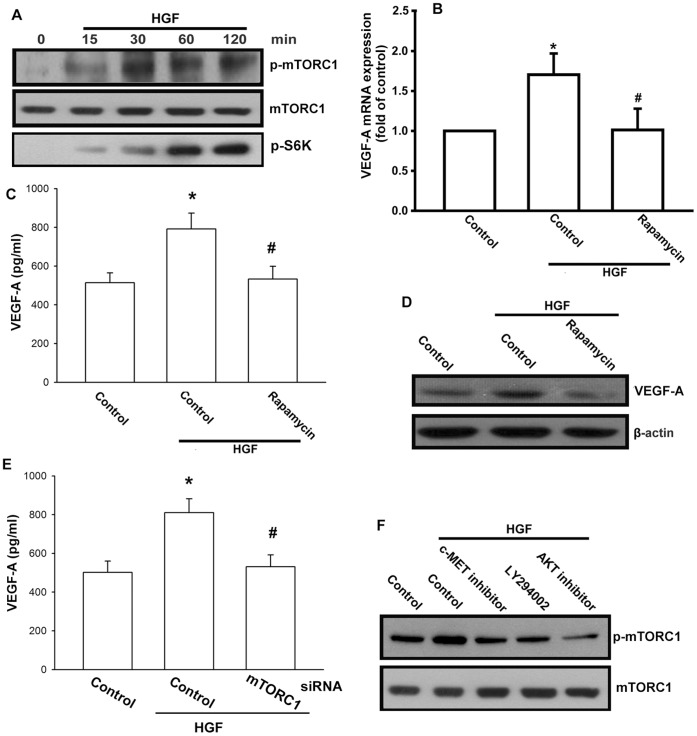
mTORC1 activation is involved in HGF-mediated VEGF-A production. (A) OASFs were incubated with HGF for indicated time intervals, mTORC1 and S6K phosphorylation was examined by Western blotting. (B–D) OASFs were pretreated for 30 min with rapamycin (30 nM) followed by treatment with HGF for 24 h, the VEGF-A expression was examined by qPCR, Western blotting, and ELISA. (E) OASFs were transfected with mTORC1 siRNA followed by stimulation with HGF for 24 h, the VEGF-A expression was examined by ELISA. (F) OASFs were pretreated for 30 min with c-Met inhibitor, Ly294002, and Akt inhibitor for 30 min, followed by stimulation with HGF for 30 min, and mTORC1 phosphorylation was determined by Western blotting. Results are expressed as the mean ± S.E. *, *p*<0.05 compared with control; #, *p*<0.05 compared with HGF-treated group.

### Quantitative Real-time PCR

Total RNA was extracted from synovial fibroblasts with a TRIzol kit (MDBio Inc., Taipei, Taiwan) and was quantified by adding 1 µl of sample to 79 µl RNase-free water. The absorbance was measured in a RNA/DNA calculator (GeneQuant Pro, GE Healthcare, Piscataway, NJ) at 260 and 280 nm. The reverse transcription reaction was performed using 2 µg of total RNA (in 2 µl RNase-free water) that was reverse transcribed into cDNA with an MMLV RT kit (Promega, Madison, WI) following the manufacturer's recommended procedures [26], [27]. The reverse transcription reaction mixture was incubated at 37°C for 60 min and then at 70°C for 5 min to inactivate MMLV. Quantitative real time PCR (qPCR) analysis was carried out with TaqMan® one-step PCR Master Mix (Applied Biosystems, Foster City, CA). cDNA template (2 µl) was added to each 25-µl reaction with sequence-specific primers and TaqMan® probes. All target gene primers and probes were purchased commercially (β-actin was used as internal control) (Applied Biosystems). qPCR assays were carried out in triplicate on a StepOnePlus sequence detection system. The cycling conditions were: 10-min polymerase activation at 95°C followed by 40 cycles at 95°C for 15 s and 60°C for 60 s. The threshold was set above the non-template control background and within the linear phase of target gene amplification to calculate the cycle number at which the transcript was detected (denoted C_T_). Reactions were normalized to copies of β-actin mRNA within the same sample using the −ΔΔCT method. The levels of mRNA are expressed as the fold change in expression level compared with that of controls.

**Figure 5:**
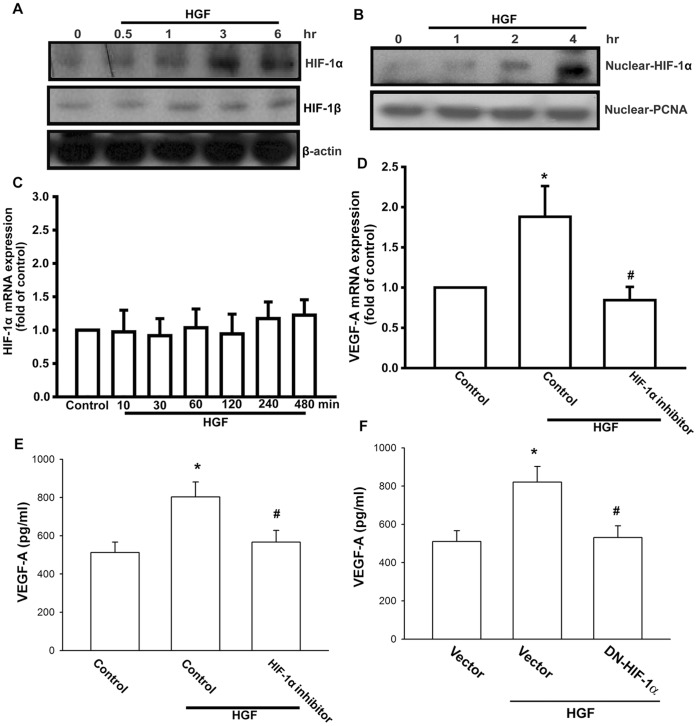
HGF enhances HIF-1α activation. (A) OASFs were incubated with HGF for indicated time intervals, and HIF-1α expression was examined by Western blotting. (B) OASFs were incubated with HGF for indicated time intervals, and nucleus HIF-1α accumulation was determent by Western blotting. (C) OASFs were incubated with HGF for indicated time intervals, and HIF-1α mRNA expression was examined by qPCR. (D&E) OASFs were pretreated for 30 min with HIF-1α inhibitor followed by treatment with HGF for 24 h, the VEGF-A expression was examined by qPCR and ELISA. (F) OASFs were transfected with HIF-1α mutant followed by stimulation with HGF for 24 h, the VEGF-A expression was examined by ELISA. Results are expressed as the mean ± S.E. *, *p*<0.05 compared with control; #, *p*<0.05 compared with HGF-treated group.

### Western Blot Analysis

Cellular lysates were prepared as described [28], [29]. Proteins were resolved using SDS-PAGE and transferred to Immobilon polyvinyldifluoride membranes. The membranes were blocked with 4% BSA for 1 h at room temperature and then probed with rabbit antibodies against human p-p85, p85, p-Akt, Akt, p-mTORC1, or mTORC1 (1∶1000) for 1 h at room temperature. After three washes, the blots were incubated with a donkey anti-rabbit peroxidase-conjugated secondary antibody (1∶1000) for 1 h at room temperature. The blots were visualized with enhanced chemiluminescence on Kodak X-OMAT LS film (Eastman Kodak, Rochester, NY).

### Transfection and Reporter Gene Assay

Human synovial fibroblasts were co-transfected with 0.8 µg HRE luciferase plasmid and 0.4 µg β-galactosidase expression vector. OASFs were grown to 80% confluency in 12-well plates and then transfected on the following day with Lipofectamine 2000 (LF2000; Invitrogen). DNA and LF2000 were premixed for 20 min and then added to the cells. After 24 h of transfection, the cells were incubated with the indicated reagents. After a further 24 h of incubation, the medium was removed, and cells were washed once with cold PBS. To prepare lysates, 100 µl reporter lysis buffer (Promega, Madison, WI) was added to each well, and cells were scraped from dishes. The supernatant was collected after centrifugation at 13,000 rpm for 2 min. Aliquots of cell lysates (20 µl) containing equal amounts of protein (20–30 µg) were placed into wells of an opaque black 96-well microplate. An equal volume of luciferase substrate was added to all samples, and luminescence was measured in a microplate luminometer. The value of luciferase activity was normalized to the transfection efficiency, which was monitored by activity of the co-transfected β-galactosidase expression vector.

### Statistics

The values reported are means ± S.E. Statistical comparisons between two samples were performed using Student’s *t-*test. Statistical comparisons of more than two groups were performed using one-way analysis of variance (ANOVA) with Bonferroni’s *post-hoc* test. In all cases, *p*<0.05 was considered significant.

**Figure 6:**
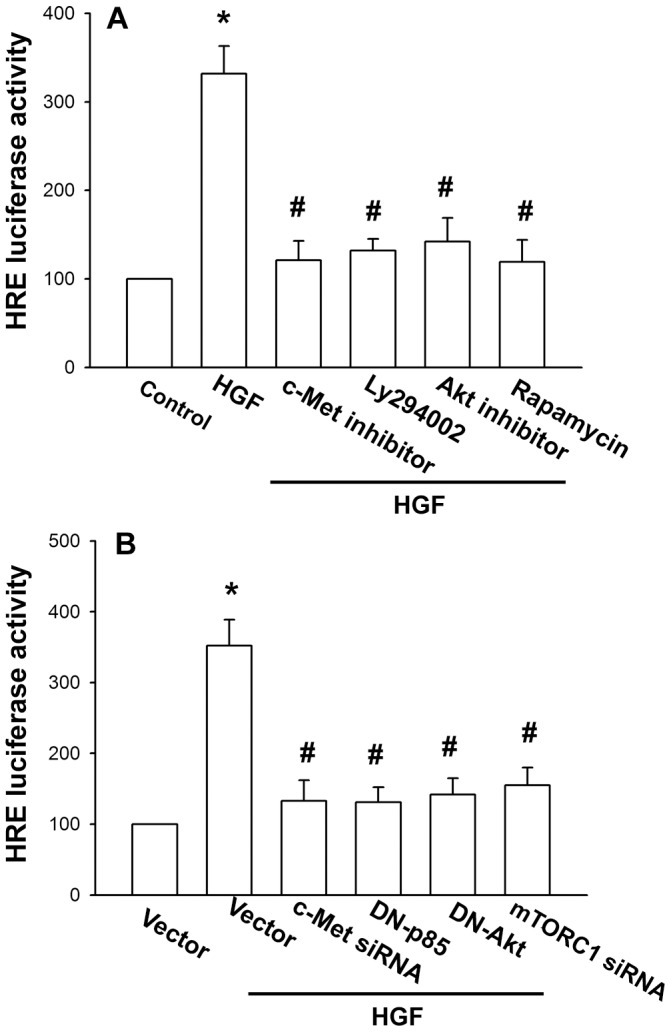
The c-Met/PI3K/Akt/mTORC1 signaling pathway is involved in the increase of HIF-1α activity in response to HGF. OASFs were pretreated with c-Met inhibitor, Ly294002, Akt inhibitor, and rapamycin for 30 min (A) or co-transfected with c-Met and mTORC1 siRNA or p85 and Akt mutant (B) before exposure to HGF. HRE luciferase activity was measured 24 h after HGF treatment, and the results were normalized to the β-galactosidase activity and expressed as the mean ± S.E. for three independent experiments performed in triplicate. Results are expressed as the mean ± S.E. *, *p*<0.05 compared with control; #, *p*<0.05 compared with HGF-treated group.

## Results

### HGF Induces VEGF-A Production in Human Synovial Fibroblasts

The typical pathology of OA includes chronic inflammation of the synovium that is characterized by infiltration of inflammatory cells and synovial hyperplasia, especially of fibroblast-like synoviocytes. Therefore, we used human synovial fibroblasts to investigate the signaling pathways of HGF in the production of VEGF-A. Treatment of OASFs with HGF (3–100 ng/ml) for 24 h induced VEGF-A mRNA expression in a concentration-dependent manner (Fig. 1A), and this induction occurred in a time-dependent manner (Fig. 1B). In addition, stimulation of cells with VEGF-A also led to increased expression of VEGF-A protein in a concentration and time-dependent manner as shown by Western blotting and ELISA assay (Fig. 1C–F). These data suggest suggesting that the HGF increased VEGF-A expression is human synovial fibroblasts.

### HGF Increases VEGF-A Production via the c-Met Receptor

It has been reported that HGF exerts its effects through interaction with a specific receptor c-Met [30]. Next, we examine whether c-Met receptor is involved in HGF-mediated VEGF-A production in human synovial fibroblasts. Transfection of cells with c-Met siRNA reduced c-Met expression in OASFs (Fig. 2A). In addition, transfection of OASFs with c-Met siRNA blocked HGF-increased VEGF-A production (Fig. 2B&C). Furthermore, c-Met inhibitor also inhibited HGF-induced VEGF-A up-regulation (Fig. 2B–D). Therefore, an interaction between HGF and c-Met is very important for VEGF-A production in OASFs.

### The PI3K, Akt, and mTORC1 Signaling Pathways are Involved in the Potentiating Action of HGF

PI3K-dependent Akt activation has been reported to regulate VEGF-A expression [31]. We next examined whether HGF stimulation also enhances PI3K/Akt activation. First, we directly measured phosphorylation of p85 in response to HGF. Stimulation of OASFs led to a significant increase in phosphorylation of p85 (Fig. 3A). Pretreatment of cells with PI3K inhibitor Ly294002 reduced HGF-increased VEGF-A production (Fig. 3B–D). Transfection with p85 mutant also reduced HGF-induced VEGF-A expression (Fig. 3E). Pretreatment of cells with c-Met inhibitor reduced HGF-mediated p85 phosphorylation (Fig. 3F). To examine the crucial role of PI3K-dependent Akt in HGF-induced VEGF-A expression, we next determined Akt Ser^473^ phosphorylation in response to HGF treatment. As shown in Figure 3A, treatment of OASFs with HGF resulted in time-dependent phosphorylation of Akt Ser^473^. Pretreatment of cells with Akt inhibitor or transfection of cells with Akt mutant antagonized HGF-induced VEGF-A expression (Fig. 3B–E). In addition, pretreatment of cells with c-Met inhibitor or Ly294002 reduced HGF-mediated Akt phosphorylation (Fig. 3G). Taken together, these results indicate that the PI3K/Akt pathway is involved in HGF-induced VEGF-A production.

Because the PI3K/Akt pathway is a major upstream activator of mTORC1, we next measured mTORC1 activation in response HGF treatment. Treatment of OASFs with HGF resulted in time-dependent phosphorylation of mTORC1 (Fig. 4A). Ribosomal S6 kinase (S6K) is major target of mTORC1 signaling. Treatment of OASFs with HGF also increased S6K phosphorylation (Fig. 4A). On the other hand, pretreatment of cells with mTORC1 inhibitor rapamycin or transfection of cells with mTORC1 siRNA reduced HGF-induced VEGF-A expression (Fig. 4B–E). In addition, incubation of cells with c-Met inhibitor, Ly294002, and Akt inhibitor also reduced HGF-mediated mTORC1 phosphorylation (Fig. 4F). Based on these results, it appears that the HGF acted through the c-Met/PI3K/Akt/mTORC1 signaling pathway to enhance VEGF-A production in human synovial fibroblasts.

### HGF Promotes HIF-1 Activation

HIF, a pivotal transcription factor, is a dominant regulator of VEGF-A expression [32]. We therefore sought to determine whether HIF was involved in HGF-induced VEGF-A expression in the OASFs. To this end, cells were treated with HGF, and the cell lysates were collected at different time intervals. The results from Western blotting indicated that HGF significantly increased protein level of HIF-1α but not HIF-1β time-dependently (Fig. 5A). Nuclear translocation of HIF-1α is necessary for its transcriptional activation of a variety of HIF-1-regulated genes [33]. We therefore used Western blotting to examine the nuclear translocation of HIF-1α protein in OASFs after HGF treatment. As shown in Fig. 5B, HGF stimulation enhanced the accumulation of HIF-1α in the nucleus in a time-dependent manner. Based on the above findings, we suggest that HGF increased the stability of HIF protein and thus the nuclear HIF-1 binding activity of HRE. We then examined whether HGF could up-regulated HIF-1α protein in OASFs via the increase of mRNA level. We found that HGF did not affect the mRNA level of HIF-1α (Fig. 5C). Pretreatment of cells with HIF-1α inhibitor antagonized HGF-increased VEGF-A production (Fig. 5D&E). In addition, the expression of VEGF-A for HGF-treated cells was found to decrease markedly after transfection with the dominant-negative mutant HIF-1α (Fig. 5F) carrying both of the deletions of the basic DNA binding domain (amino acids 4–27) and the carboxyl-terminal transactivation domain (amino acids 390–826), thus effectively inhibiting HIF-1α activity [34]. Based on these findings, we suggest that HGF enhances the stabilization and DNA binding activity of HIF-1α.

### HGF-induced HIF-1 Activation and Subsequent VEGF-A Expression via c-Met, PI3K, Akt, and mTORC1 Pathways

We further explored whether c-Met, PI3K, Akt, and mTORC1 pathways were involved in the HGF-induced HIF-1α activation in the cultured OASFs. The HGF mediated increase of HRE promoter activity was inhibited by c-Met inhibitor, Ly294002, Akt inhibitor, and rapamycin (Fig. 6A) or c-Met and mTORC1 siRNA or p85 and Akt mutant (Fig. 6B). Therefore, c-Met, PI3K, Akt, and, mTORC1 signaling pathways are involved in HGF-mediated HIF-1α activation.

## Discussion

OA is a heterogeneous group of conditions associated with defective integrity of articular cartilage as well as related changes in the underlying bone. Neovascularization, the formation of new blood vessels, can maintain the chronic inflammatory status by transporting the inflammatory cells to the site of synovitis as well as supplying nutrients and oxygen to pannus [35], [36]. VEGF-A is a major angiogenic factor in OA joints [37]. In addition, HGF plays important role during OA pathogenesis. However, the effect of HGF on VEGF-A expression in human synovial fibroblasts is mostly unknown. Here, we found VEGF-A as a target protein for the HGF signaling pathway that regulates the neovascularization. We showed that potentiation of VEGF-A by HGF requires activation of the c-Met, PI3K, Akt, mTORC1, and HIF-1α signaling pathways.

PI3K may possibly regulated the cell function by promoting the phosphorylation of Akt on Ser^473^ and its downstream pathways of mTORC1 [38]. Our results demonstrated that pretreatment of OASFs with PI3K, Akt, or an mTORC1 inhibitor antagonized the HGF-induced increase of VEGF expression. On the other hand, HGF treatment increased the level of mTORC1 phosphorylation. This effect was inhibited by Ly294002 and Akt inhibitor, indicating the involvement of PI3K/Akt-dependent mTORC1 activation in HGF-mediated VEGF expression. In addition to VEGF expression and angiogenesis, a similar signaling pathway has also been reported in N-myc induced VEGF expression and angiogenesis in neuroblastoma, which involved PI3K-dependent Akt, and mTORC1 activation [39]. Regulation of angiogenesis and tumor growth by hispidulin is also related to PI3K/Akt/mTORC1 signal cascade [40]. Notoginsenoside Ft1 promoted VEGF expression and angiogenesis, which involved PI3K/Akt, and mTORC1 transactivation [41]. Taken together, these results show that the PI3K/Akt/mTORC1 may be a common route for VEGF expression and angiogenesis.

HIF-1 is thought to play a major role in VEGF-A expression [32]. HIF-1α has been reported to activate VEGF-A expression by binding to the HRE site within the VEGF-A promoter in response to hypoxia [42]. Likewise, the activation of HIF-1α by HGF also resulted in an induction of VEGF-A transcription activity through the HRE site in OASFs, although many other possible response elements, including activator protein-2, nuclear factor-κB, and simian virus 40 promoter factor 1, are located within the VEGF promoter. In this study, HIF-1α inhibitor and mutant complete blocked HGF-mediated VEGF expression. OASFs up-regulate HIF-1α after HGF treatment in a time-dependent manner. It is apparent that HGF-induced VEGF-A expression is substantially mediated by the HRE. This is not the first study to provide evidence that HGF is increased through HIF-1α transactivation. In the hepatoma cells, HGF promoted gene expression by increasing HIF activity [43], and in the HGF-induced survival in carcinoma cells, involved HIF-1α activation [44]. In concert with our study, other studies would seem to suggest that HGF may mediate HIF-1α activation in many gene expressions and cell functions. However, we still cannot rule out the effects of other transcription factors in HGF-induced VEGF-A production in OASFs.

Factors that increase the expression of VEGF have been suggested as potential therapeutic targets to delay or reduce the joint destruction that occurs in arthritis patients [45]. Based on the *in vitro* effect of HGF on VEGF expression, HGF may be a potential target for blockage of VEGF expression. However, further studies are needed to better understand the factors that control the expression of VEGF in the joint fluid of OA patients. This knowledge may open new doors to treatment and lead to the inhibition of the pathological processes of OA.

In conclusion, we explored the signaling pathway involved in HGF-induced VEGF-A expression in human synovial fibroblasts and found that HGF increased VEGF-A expression through c-Met receptor and activation of PI3K, Akt, mTORC1, and HIF-1α pathways in OASF. These findings may provide a better understanding of the mechanisms of OA pathogenesis.
